# Heat Waves Change Plant Carbon Allocation Among Primary and Secondary Metabolism Altering CO_2_ Assimilation, Respiration, and VOC Emissions

**DOI:** 10.3389/fpls.2020.01242

**Published:** 2020-08-14

**Authors:** Christiane Werner, Lukas Fasbender, Katarzyna M. Romek, Ana Maria Yáñez-Serrano, Jürgen Kreuzwieser

**Affiliations:** ^1^ Ecosystem Physiology, University of Freiburg, Freiburg, Germany; ^2^ Center of Ecological Research and Forest Applications (CREAF), Universitat Autònoma de Barcelona, Barcelona, Spain; ^3^ Global Ecology Unit CREAF-CSIC-UAB, Cerdanyola del Vallès, Barcelona, Spain

**Keywords:** carbon allocation, day respiration, temperature stress, ^13^C position-specific labeling, photosynthesis, pyruvate, dark, volatile organic compounds (VOC)

## Abstract

Processes controlling plant carbon allocation among primary and secondary metabolism, i.e., carbon assimilation, respiration, and VOC synthesis are still poorly constrained, particularly regarding their response to stress. To investigate these processes, we simulated a 10-day 38°C heat wave, analysing real-time carbon allocation into primary and secondary metabolism in the Mediterranean shrub *Halimium halimifolium L*. We traced position-specific ^13^C-labeled pyruvate into daytime VOC and CO_2_ emissions and during light-dark transition. Net CO_2_ assimilation strongly declined under heat, due to three-fold higher respiration rates. Interestingly, day respiration also increased two-fold. Decarboxylation of the C1-atom of pyruvate was the main process driving daytime CO_2_ release, whereas the C2-moiety was not decarboxylated in the TCA cycle. Heat induced high emissions of methanol, methyl acetate, acetaldehyde as well as mono- and sesquiterpenes, particularly during the first two days. After 10-days of heat a substantial proportion of ^13^C-labeled pyruvate was allocated into *de novo* synthesis of VOCs. Thus, during extreme heat waves high respiratory losses and reduced assimilation can shift plants into a negative carbon balance. Still, plants enhanced their investment into *de novo* VOC synthesis despite associated metabolic CO_2_ losses. We conclude that heat stress re-directed the proportional flux of key metabolites into pathways of VOC biosynthesis most likely at the expense of reactions of plant primary metabolism, which might highlight their importance for stress protection.

## Introduction

Global climate change will markedly alter environmental conditions for plant growth and functioning. However, potential changes between the magnitudes of plant carbon sequestration by photosynthesis relative to the rate of growth, respiration and secondary metabolism are highly uncertain. There is a marked disparity between the process-based formulations of biochemical models of photosynthetic CO_2_ assimilation (Farquhar et al., 1980), and models of respiration (Sweetlove et al., 2013; O’Leary et al., 2019), allocation (Brüggemann et al., 2011), or those describing the response of the secondary metabolism to resources availability (e.g., Larbat et al., 2016). C-allocation is thought to be driven by the C balance between supply (source) and demand (sink), however, the dominating factor is still a matter of debate (Körner, 2013; Fatichi et al., 2014). Indeed, plants exhibit a higher than expected plasticity to adjust both source and sink capacities in response to environmental changes (e.g., Wegener et al., 2015b), or carbon allocation into primary and secondary metabolism (e.g., Heinrich et al., 2015). In particular, knowledge on plant internal readjustment of the metabolic carbon fluxes in response to environmental stresses for synthesis of defence compounds such as volatile organic compounds (VOCs) is still limited (e.g., Huang et al., 2019). In the light of extreme climatic events, such as drought spells and heat waves, the plant’s ability to rapidly adjust its metabolism and protect the photosynthetic machinery against these stresses will be decisive for its persistence. Plant species, particularly from hot and arid environments, have evolved multiple structural and functional adaptations to withstand environmental stress (Werner et al., 1999; Werner et al., 2002). However, heat waves, i.e., the rapid occurrence of excessive temperatures over prolonged periods, can expose plants and ecosystems to stress levels beyond their acclimation capacity (Teskey et al., 2015; Tatarinov et al., 2016). Over the past decades, record-breaking monthly temperature extremes and heat waves have increased (Coumou et al., 2013; IPCC, 2014), strongly impacting ecosystem carbon balances (Ciais et al., 2005; Bastos et al., 2013). As heat waves are predicted to further increase in frequency and intensity (Perkins-Kirkpatrick and Gibson, 2017) it is imperative to understand how they will affect plant physiological processes and growth (Teskey et al., 2015).

Plants have evolved a plethora of mechanism to cope with heat stress, which causes production of reactive oxygen species (ROS) in plant cells (Jajic et al., 2015; Dietz et al., 2016; Suzuki and Katano, 2018; Waszczak et al., 2018). Scavenging of ROS can be achieved through elevated levels of antioxidants, accumulation and adjustment of solutes, specialized kinase cascades, as well as chaperone signalling (see review by Dietz et al., 2016). Another mechanism to mitigate the effects of oxidative stress under high temperatures is through the production of volatile isoprenoids (Vickers et al., 2009), which have been shown to reduce the levels of damaging ROS within the leaf (Loreto and Velikova, 2001; Sharkey and Yeh, 2001; Affek and Yakir, 2002; Affek and Yakir, 2003; Sharkey, 2005). Elevated temperatures increase the fluidity of membranes and can induce leakiness. Volatile isoprenoids such as isoprene and/or monoterpenes have often been shown to increase heat resistance, most probably by stabilizing membranes, in particular the thylakoid membranes (Singsaas et al., 1997; Sharkey and Yeh, 2001; Behnke et al., 2007; Sharkey et al., 2008). Recent reports have shown that this may be an indirect effect mediated *via* modulating membrane bound proteins of the thylakoid membranes (Harvey et al., 2015). Moreover, VOCs can aid to enhance the photosynthetic quenching of excess energy in the thylakoid membranes (Penuelas and Munne-Bosch, 2005; Pollastri et al., 2014), which is particularly critical to protect the photosynthetic apparatus (Rennenberg et al., 2006; Niinemets, 2018). Consequently, many plant species enhance the release of isoprene and monoterpenes under heat stress (Loreto and Schnitzler, 2010), which can be mediated by enhanced *de novo* biosynthesis and/or increased vapour pressure of stored compounds (Peñuelas and Staudt, 2010; Holopainen et al., 2018). It must be denoted, that severe heat stress has also been shown to reduce both *de novo* and total emissions of mono- and sesquiterpenes (Kleist et al., 2012).

*De novo* synthesis of VOCs depends on the availability of carbon, as well as energy provided by primary metabolism. Therefore, the availability of building blocks has a major impact on the concentration of any secondary metabolite, demonstrating the high degree of connectivity between primary and secondary metabolism (Dudareva et al., 2013). Carbon partitioning occurs at the metabolic branching points of the respiratory pathways and secondary metabolism. The relevant pathways are linked *via* a number of key metabolites including the central intermediates pyruvate (Werner and Gessler, 2011), phosphoenolpyruvate (PEP) or malate (Souza et al., 2018). Notably, pyruvate is a known substrate in a large array of secondary pathways leading to the biosynthesis of many VOCs, such as isoprenoids, some oxygenated VOCs, aromatics as well as fatty acid oxidation products, which can be emitted by plants (Jardine et al., 2014). However, elevated temperatures also increase respiratory losses (Atkin, 2003) and reduce carbon assimilation, which may limit the availability of carbon skeletons for secondary metabolism including biosynthesis of volatile compounds.

In general, leaf day respiration is recognised to play a central role to provide carbon backbones for several metabolic processes (Tcherkez et al., 2012). Due to the light-inhibition of the respiratory pathways (Atkin et al., 1998), day respiration provides a low-flux though highly dynamic metabolic pathway that interacts with photosynthesis and environmental conditions (Tcherkez et al., 2017). While the mitochondrial respiratory tricarboxylic acid (TCA) cycle is partially inhibited in the light (e.g., Sweetlove et al., 2010; Tcherkez et al., 2012), there are several metabolic processes both in the cytosol and organelles causing partial decarboxylation of pyruvate. Thus, while the C2-C3 moiety of pyruvate fuels anabolic processes such as fatty acid and secondary compound synthesis, the carboxyl group at the C1 position is released as CO_2_ (Werner and Gessler, 2011). However, few studies have investigated carbon allocation into day respiration in response to rising temperature and VOC emissions (Fares et al., 2011) and even less is known regarding the response to heat extremes.

We hypothesise that heat stress leads to enhanced carbon allocation into secondary metabolism in particular VOC biosynthesis for stress-protection and that such metabolic re-adjustment may result in higher CO_2_ release in the light. Moreover, we assume a trade-off between carbon investment into protective functions such as VOC synthesis and maintenance of a positive carbon balance under severe stress, particularly if stress levels exceed the acclimation potential during extreme events.

To test this hypothesis, we selected the highly stress-adapted Mediterranean shrub, *Halimium halimifolium* L. (Zunzunegui et al., 2000; Peperkorn et al., 2005; Werner et al., 2010), a member of the Cistaceae family, which is widely spread in Mediterranean shrublands (Thanos et al., 1992). Species of this family are particularly suitable to test our hypothesis since they are characterized by a rich secondary metabolism forming an immense quantity and diversity of plant secondary metabolites including a rich volatile emission blend (Kesselmeier and Staudt, 1999; Peñuelas and Llusià, 2001; Owen et al., 2002; Rivoal et al., 2010; Bracho-Nunez et al., 2013; Papaefthimiou et al., 2014; Haberstroh et al., 2018; Yáñez-Serrano et al., 2018). In addition, *H. halimifolium* shows large amplitudes in daytime δ^13^CO_2_ release (Werner et al., 2009; Wegener et al., 2010; Wegener et al., 2015a), which most likely are connected to processes of plant secondary metabolism (Priault et al., 2009; Werner and Gessler, 2011; Lehmann et al., 2016). Such properties suggest that members of the Cistaceae family possess a high capability to cope with heat by up-regulating processes of the secondary metabolism, thus, redirecting cellular C fluxes. Cistaceae possess trichomes which secrete a resinous exudate (Papaefthimiou et al., 2014) and can store substantial amounts of VOCs in secretarial trichomes (Gulz et al., 1996). A study in France has shown that local VOC emissions from Mediterranean shrublands can be in the same order of magnitude of non-methane VOC emissions from anthropogenic sources, and thus can exert a significant impact on air quality (Simon et al., 2006). Cistus species are predicted to be favoured by climate change due to their high plasticity to cope with environmental stresses (Correia and Ascensão, 2017). However, extreme climatic events, such as sudden heat waves or severe drought, may exceed the acclimation potential and induce cascading effects on species and ecosystem functioning (Caldeira et al., 2015). Such extreme climatic events are predicted to increase both in intensity and frequency in Mediterranean regions (Seneviratne et al., 2012).

The objective of our study was to investigate potential changes in leaf internal carbon allocation, i.e., carbon assimilation, respiration and investment into VOC under extreme heat stress. In particular, we aimed to investigate whether plants under severe stress would still invest into *de novo* synthesis of VOCs and used position-specific ^13^C-labeled pyruvate experiments to trace ^13^C-allocation into VOCs *versus* CO_2_ in light and dark. To explore these effects, we subjected *H. halimifolium* to an extreme 10-day heat wave of 38°C.

## Material and Methods

### Plant Material and Labeling Experiments

Mediterranean drought-adapted, semi-deciduous *Halimium halimifolium* L. plants (Zunzunegui et al., 2002; Wegener et al., 2015a) were grown from seeds in a greenhouse for three years. Plants that reassemble a genetic variability were grown under similar conditions in a semi-controlled greenhouse, with natural light and additional illumination (12 h); temperatures were regulated by heating during winter. Plants were cultivated in 3 L plastic pots on a potting soil/sand (2/1, v/v) mixture. They were fertilised weekly with ¼-strength modified Hoagland’s fertilizer solution (Peperkorn et al., 2005). Four weeks before the experiments, plants were subdivided into two groups and placed into two walk-in chambers to allow for acclimation at day/night conditions of 25/20°C, 60/60% relative humidity and 14/10 h light/dark with 900/0 µmol m^-2^ s^-1^ photosynthetic active photon flux density. Plants were watered according to their demand. To simulate the heat wave, on day 1 the temperature of one chamber was stepwise increased in 2°C/h steps over 8 h up to 40°C and thereafter kept constant at 38°C during day and night for ten days of continuous measurements and subsequent labeling experiments.

Branches of six plants were placed into self-constructed 600 ml borosilicate glass cuvettes (Kummer, Freiburg, Germany) two days before the heat treatment. The bottomless cuvettes were closed with PTFE-foil at the plant stem and ventilated by small fans; an additional empty cuvette was kept as reference. The cuvette temperature increased by 0.40 ± 0.12°C during the light period compared to the dark period. The cuvette position was adjusted to maintain the natural orientation of the branches. Absolut humidity of the air stream was maintained constant but due to the increase in temperature during the heat treatment relative humidity in the cuvettes decreased at 38°C. Fluxes of VOCs, H_2_O and CO_2_ and their isotopic composition were continuously recorded prior and during the heat treatment.

For labeling experiments, six plants were placed into cuvettes an evening before the measurements. A total of 36 plants were used; half of them were kept under control temperatures, half were consecutively exposed to a 10-day heat treatment. After 10-days of heat or control temperature, branches were carefully cut and re-cut immediately under water to avoid xylem embolism. The deionized water was replaced by a 10 mM ^13^C-labeled pyruvate solution after 10 min. We applied position-specific ^13^C-labeled pyruvate 99% ^13^C-enriched at the C1 or the C2 position (Cambridge Isotope Laboratories, Andover, MA, USA). Continuous measurements of the isotopic composition of VOCs and ^13^CO_2_ as well as photosynthetic gas exchange fluxes were recorded for 60 min in the light, after which the plants were darkened for 30 min. labeling procedure and concentrations had been established for *H. halimifolium* by Priault et al. (2009) and Lehmann et al. (2016) to ensure that no changes in physiological processes occurred. Plants, which did respond with stomatal closure at any stage of the treatment, were excluded from the analysis.

### On-Line Measurement System for Plant VOC, ^13^CO_2_, and H_2_O Emissions

The measuring system is described in detail in Fasbender et al. (2018). Briefly, it consists of (i) a zero air generator, providing ultra-pure hydrocarbon free air with controlled CO_2_ and H_2_O concentrations, (ii) the cuvette system to capture trace gas emissions of plants, and (iii) the analytical section for real-time detection of VOC, CO_2_ and H_2_O fluxes and their ^13^C isotopes consisting of a proton transfer reaction time-of-flight mass spectrometer (4000 ultra, PTR-TOF-MS, Ionicon Analytic, Innsbruck, Austria), an isotope ratio infrared spectrometer (IRIS, Thermo Fisher Scientific, Bremen, Germany), and an infrared gas analyser IRGA (LI-7000 CO_2_/H_2_O Analyser; LI-COR, Lincoln, NE, USA).

The inlet flow of the plant cuvettes was controlled by mass flow controllers (Omega Engineering, Stamford, CT, USA) at 500 ml min^‑1^ resulting in a calculated residence time in the cuvette of 4.8 min. The response-time of air leaving the cuvette and reaching the PTR-TOF-MS and IRIS/IRGA was less than 4 and 10 s, respectively. All cuvette parts were made of glass or PFA, and outlet lines were continuously isolated and heated (60°C) to prevent VOC adsorption or water condensation.

### VOC Detection by PTR-TOF-MS

The PTR-TOF-MS was operated at 2.7 mbar drift pressure, 600 V drift voltage, at an E/N of 120 Td, and drift tube heated to 80°C. H_2_O^+^ and O_2_^+^ ions in the drift tube were kept lower than 5% with respect to the hydronium ions. The mass resolution was 2,000 ± 500 m/Δm depending on the compound. Non-^13^C-labeled VOCs were detected at m/z 45.03 for acetaldehyde, 75.04 for methyl acetate, 137.13 for monoterpenes and 205.20 for sesquiterpenes; their isotopologues containing one ^13^C-atom were detected at m/z +1. Calibration of the assigned VOCs was done using the Liquid Calibration Unity (LCU, Ionicon Analytic, Innsbruck, Austria) either with a multicomponent calibration gas standard for acetaldehyde and monoterpenes (1,000 ppb ± 5%, Ionicon Analytic, Innsbruck, Austria) or liquid standards for sesquiterpenes and methyl acetate (Sigma-Aldrich, Taufkirchen, Germany). For acetaldehyde and monoterpenes humidity dependent calibrations were performed. For methyl acetate liquid calibrations were carried out with water-based solutions while for sesquiterpenes hexane-based solutions were used. PTR-TOF-MS data were post-processed by correction for non-extending and extending dead times as well as the correction for Poisson statistics (Titzmann et al., 2010), iterative residual analysis and cumulative peak fitting (Müller et al., 2013) using the PTR-TOF Data Analyser software (version 4.49). Data was normalized to primary ions and water, background subtracted, and calibration factors applied. Data from PTR-TOF-MS was synchronized with IRIS and IRGA data. Emission rates were calculated considering the background corrected VOC concentrations in the cuvettes, leaf area and flow rates.

### ^13^CO_2_ Detection by IRIS and IRGA

^13^CO_2_ fluxes [nmol m^-2^ s^-1^] were calculated from the differences in ^13^CO_2_ isotopic composition and their concentrations between empty and plant-containing cuvettes derived from IRIS. The IRIS measures the ^12^CO_2_ and ^13^CO_2_ fluxes continuously. Calculating the net ^13^CO_2_ flux enables to trace both, the natural ^13^C- isotope discrimination during photosynthetic CO_2_ uptake and the ^13^CO_2_ release from metabolic processes after labeling.

Net fluxes of ^13^CO_2_ (*e_13CO2_*) were calculated per projected leaf area (*s*) as:

$$e_{13CO_{2}}=\frac{u_{in}}{s}*{(}^{13}c_{out}{-}^{13}c_{in})$$

Where *u_in_* is the molar flow of incoming air calculated as:

$$u_{in}=\frac{V}{t}*\frac{p}{R*T}$$

where: *V* - gas volume, *t -* time, *p* - gas pressure, *R* - ideal gas constant, *T* - temperature, and *^13^c_out_/^13^c_in_* is the molar fraction of ^13^CO_2_ at the inlet and outlet of the cuvette calculated as:

c13=((δ13CO21000+1)∗(c13c12)VPDB((δ13CO21000+1)∗(c13c12)VPDB)+1)∗ctotal

where: *c_total_* - mole fraction of total CO_2_ in the cuvette and *(^13^C/^12^C)_VPDB_*- isotope ratio of the international standard *Vienna Pee Dee Belemnite*.

Calibration for isotopic composition was performed automatically whenever any significant change in the CO_2_ mixing ratio in the sample gas occurred, e.g., during the change between plant and blank cuvette. Furthermore, automatic calibration for concentration dependency of the analyser was conducted nightly.

Transpiration (*E*) and assimilation rates (*A*) were calculated according to Caemmerer and Farquhar (1981). The IRGA was calibrated weekly with synthetic air (0 and 408 ppm; Messer, Bad Soden, Germany) for CO_2_ and right before the experiment for H_2_O by a manufacture calibrated GFS3000 (Walz GmbH, Effeltrich, Germany).

### Chlorophyll a Fluorescence

Chlorophyll a fluorescence was measured with the MINI-PAM II fluorometer (Walz, Effeltrich, Germany). The maximum quantum yield of PSII was measured after dark-adapting leaves with leaf clips for 20 min (Werner et al., 2002). A saturation pulse of 10.000 µmol m^-^² s^-1^ was applied; measuring light intensity, gain and damping factors were adjusted to allow optimal pulse resolution. Three leaves per plant were measured on 20 control and 20 heat stressed plants (after 10 days of heat exposure). In a few cases, not all three readings per plant could be used due to leaf damage or too low fluorescence signals.

### Statistics

Data were tested for equal variance and normality distribution by a Shapiro-Wilk test. A repeated measurement ANOVA with a Holm-Sidak post-hoc test was applied to test for significant changes in total net carbon assimilation, nocturnal respiratory losses and net carbon balance during the 10-day heat waves.

## Results

**Figure 1:**
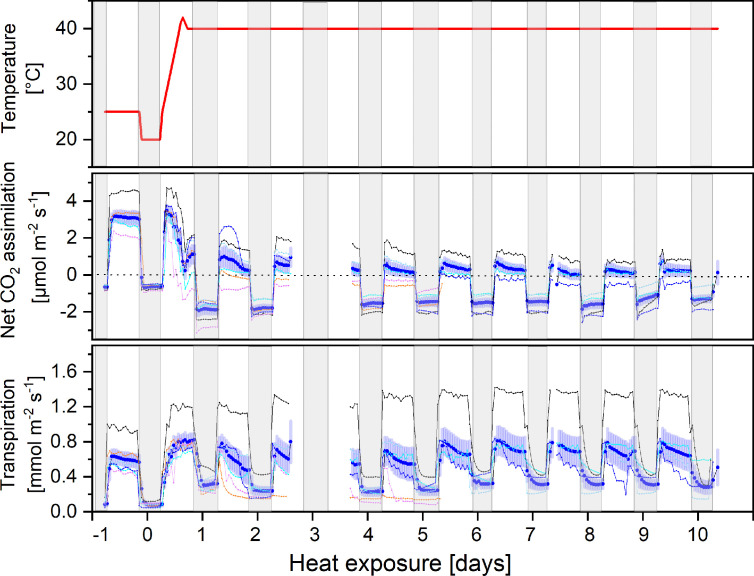
Diurnal courses of air temperature in the climate chamber, net CO_2_ assimilation, and transpiration rates (upper, mid and lower panel respectively). Plants were continuously measured under controlled conditions (day -1) and 10 days in the heat (38°C). Mean values of 6 plants (solid blue line) and standard error and their individual fluxes are shown. Individual fluxes are colour-coded from reddish to dark blue, ranking individuals from the most to the least affected plants by heat, respectively. Two dying individuals had to be replaced on day 6. Data gaps are due to an instrument failure on day 3 and a calibration routine on day 8. Gray shading indicates night periods.

Exposure to continuous heat (38°C) for 10 days considerably reduced net CO_2_ assimilation compared to pre-treatment rates, while nocturnal respiration increased significantly (p < 0.001, **Figure 1**). The strongest depression of net CO_2_ assimilation occurred during the first two days. Thereafter, net assimilation decreased slowly, but steadily, further lowering carbon gain during the light period compared to the respiratory losses at night. Notably, the amplitude between rates of CO_2_ release during night and uptake during day remained widely constant (**Figure 1**), due to an immediate 3.2-fold increase in nocturnal respiration. Interestingly, mean nocturnal respiration slowly decreased with increasing stress duration, being only 2.3-fold higher than under control conditions at the end of the heat stress. Internal CO_2_ concentrations increased in all plants under heat stress from 256 ± 14 to 354 ± 57 ppm.

There was a striking phenotypic heterogeneity among plant individuals: while some individuals showed a strong stress response resulting in negative net CO_2_ balances within the first two days (reddish colours in **Figure 1**), others still maintained positive assimilation rates during the day throughout the whole period (two individuals had to be replaced on day 6). Mean transpiration slightly increased in response to heat treatment, but again showing high variability between plant individuals (**Figure 1**). Though plants were kept well-watered, they responded with stomatal closure during the heat period (data not shown) most probably in response to a drop in relative humidity due to the higher temperatures. Surprisingly, nocturnal transpiration seemed to be enhanced in heat stressed plants.

Beside lower stomatal conductance and enhanced respiration, also a direct inhibition of the photosynthetic light use efficiency was observed because the maximum quantum yield of photosystem II declined from 0.752 ± 0.05 under control conditions to 0.57 ± 0.12 (n=20) after 10 days of heat.

**Figure 2:**
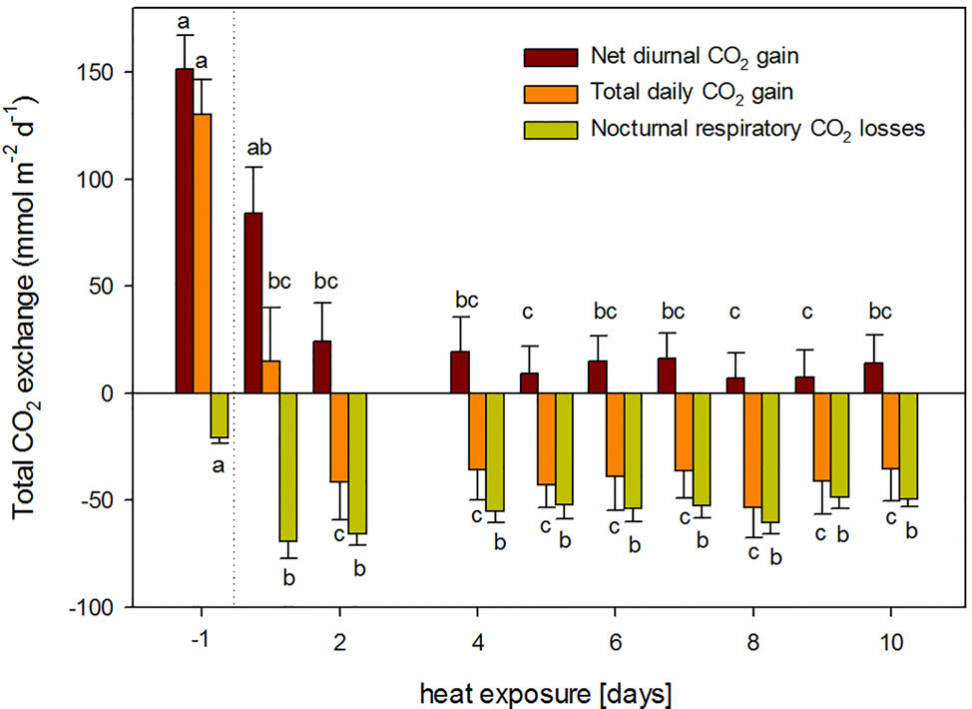
Total daily CO_2_ exchange during the heat treatment: total diurnal net CO_2_ assimilation (dark red); total nocturnal respiratory CO_2_ losses (yellow); and total daily net carbon balance (orange) for the pre-treatment conditions (day -1, 25°C) and during 10-day continuous heat exposure (38°C). Measurements on day three are lacking due to an instruments failure. Mean of n = 6 and SE of plants shown in **Figures 1**.

Plant were not able to maintain a positive net carbon balance during the 10-day heat exposure (**Figure 2**). Diurnal net assimilation was significantly reduced by 44% and 84% during the first and second day of heat, respectively. Moreover, nocturnal respiration increased significantly 3.3-fold. Therefore, total plant carbon balance turned negative after day three (**Figure 2**). Even though total nocturnal respiratory CO_2_ losses decreased with stress duration, it could not counterbalance the concomitant decline in daily net assimilation. Thus, plants exhibited a negative carbon balance throughout the rest of the heat treatment (**Figure 2**).

**Figure 3:**
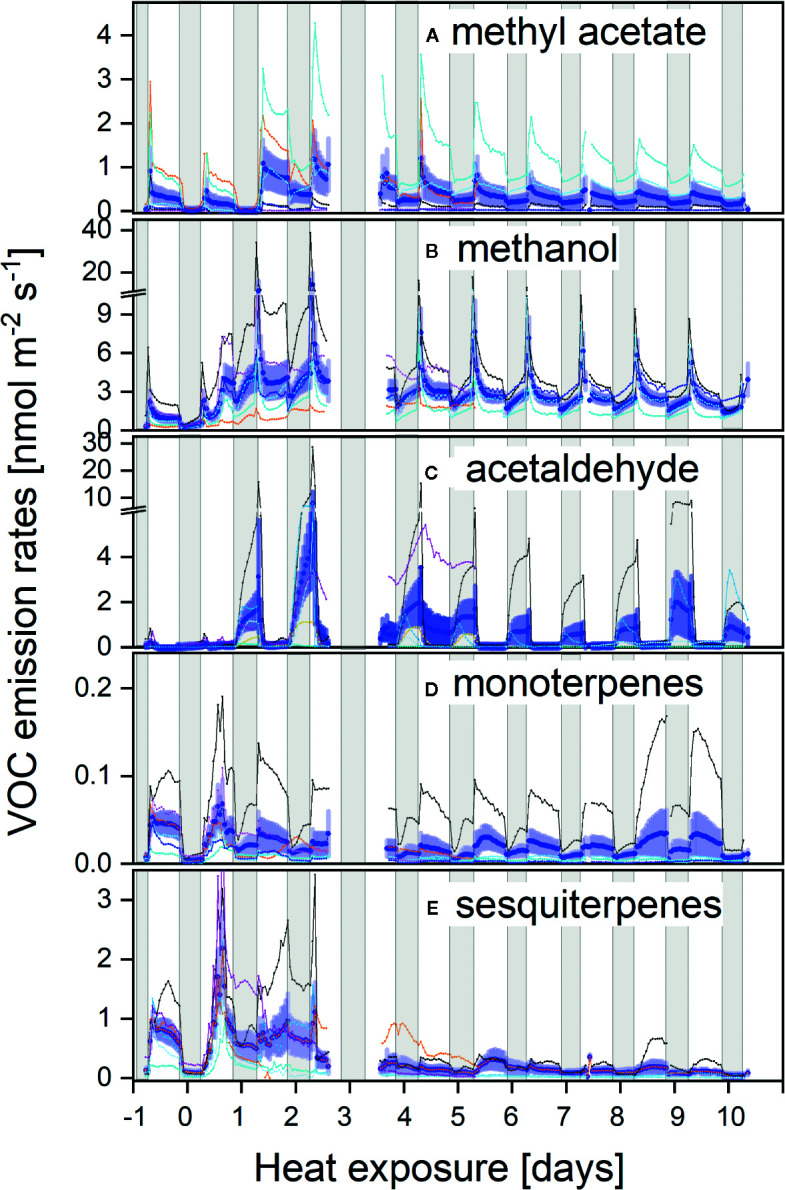
**(A–E)** Diurnal courses of VOC fluxes prior and during the heat stress of methyl acetate **(A)**, methanol **(B)**, acetaldehyde **(C)**, monoterpenes **(D)**, and sesquiterpenes **(E)**. Individual fluxes are colour-coded as in **Figure 1** from reddish to dark blue, ranking individuals from the most to the least affected plants by heat, respectively.

The strong phenotypic variability between plant individuals was also reflected in the heat response of VOC emissions regarding both, intensity and chemo-diversity. Overall, the strongest emissions occurred as an immediate stress response within the first days of heat exposure. Large fluxes were observed for methyl acetate (**Figure 3A**), methanol (**Figure 3B**), acetaldehyde (**Figure 3C**), as well as monoterpenes (**Figure 3D**) and sesquiterpenes (**Figure 3E**). Sesquiterpenes and to a lesser extent monoterpenes showed the most pronounced reaction upon the initial increase in temperature, resulting in large fluxes during the first two days. Subsequent low sesquiterpene emission rates may indicate that sesquiterpene pools became depleted or biosynthesis decreased. Monoterpene emission first decreased but increased again after four days of stress exposure, even though total monoterpene emissions were relatively low in this species. Only plant individuals with very low net CO_2_ assimilation (red lines **Figures 1** and **3**) maintained higher sesquiterpene fluxes during the following days but without a clear light-dependent pattern.

Interestingly, methyl acetate, methanol, and acetaldehyde emissions exhibited pronounced morning peaks upon stomatal opening during the initial days of the heat wave (**Figures 3A–C**). During the course of the experiment, methyl acetate emissions remained high during the day and even an elevated night-time flux could be observed. Surprisingly, besides the morning burst, highest acetaldehyde fluxes were always observed during night in the heat treatment, while fluxes were negligible prior to stress application (**Figure 3C**).

Large phenotypic plasticity between plant individuals was also reflected in a pronounced chemodiversity regarding their emission pattern: for example, the individual with the highest methyl acetate flux (**Figure 3B**, light blue line) was a low mono- and sesquiterpene-emitter. Strongly stressed plants as seen from negative CO_2_ balances (**Figures 1** and **3**, red lines) revealed higher VOC emission rates, particularly for mono- and sesquiterpenes but also for methyl acetate. Still, the highest monoterpene emitter was among the plant individuals maintaining highest net CO_2_ assimilation rates under heat stress (**Figures 1B** and **3E**; thin black line). Thus, these continued real-time emission pattern of CO_2_ and VOCs indicated a high dynamic response to heat exposure, as well as a high chemodiversity of emission blends between individuals.

**Figure 4:**
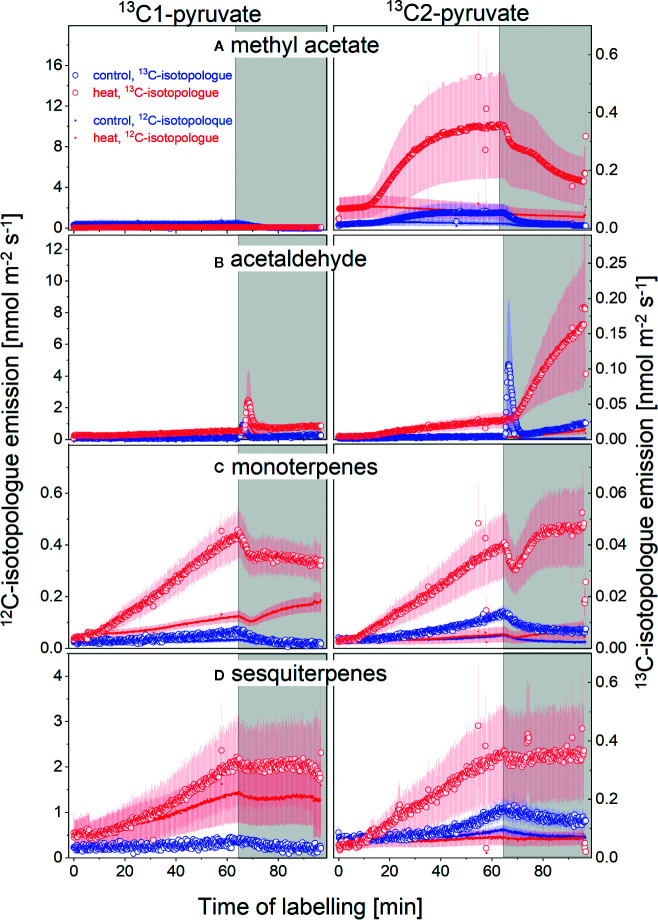
^13^C-VOC labeling by positions specific ^13^C-pyruvate during light-dark transitions. Emissions of the parent (^12^C-isotopologue, solid line, left scale) and the first isotope (^13^C-isotopologue, open symbols, right scale) of **(A)** methyl acetate, **(B)** acetaldehyde, **(C)** monoterpenes, and **(D)** sesquiterpenes. Plant of the control (blue) and 10-day heat treatment (red) fed with ^13^C1-pyruvate (left panels) or ^13^C2-pyruvate (right panels) for 1 h after which plants were darkened for 30 min. N = 4-8, standard errors are given. Please note that scales differ for each compound; left and right hand scales are set to match the proportional changes in the ^12^C and ^13^C-isotoplogues for each compound, i.e., the ^13^C-scale is set to reflect the changes in the first natural ^13^C- isotopologue abundance (m/z+1) proportional to the parent (mz), which is an order of magnitude higher. An increase in the ^13^C-isopologue emission compared to the parent does therefore indicate label incorporation into this compound.

To elucidate to what extend VOC emissions were sustained by *de novo* synthesis or release from storage pools, we conducted position-specific ^13^C-labeling experiment by feeding the branches with pyruvate labeled at the C1 or C2 position (**Figure 4**, left and right hand panels, respectively) in control plants and after 10 days of heat exposure (blue and red lines, respectively). The y-axes scales in **Figure 4** are set to reflect proportional changes in the parent mass (left y-axes) and the first heavy isotopologue (right y-axes) for each compound. Very clearly, neither heat stressed nor control plants did incorporate detectable amounts of the ^13^C1-atom of pyruvate into methyl acetate (**Figure 4A**, left panel). In contrast, the ^13^C2 atoms of pyruvate were efficiently used for biosynthesis of methyl acetate, which is visible by an increase in ^13^C-emission (**Figure 4A**, right panel). Moreover, heat stressed plants used almost 5-times more ^13^C2-pyruvate than non-stressed control plants (compare red with blue lines, **Figure 4A**). We also observed very interesting labeling patterns for acetaldehyde. Similar to methyl acetate, the heat-stressed and control plants did show little ^13^C1-incorporation of pyruvate for acetaldehyde biosynthesis (left panel, **Figure 4B**). However, heat stressed plants very strongly incorporated the ^13^C2-atom into acetaldehyde, an effect not seen in control plants (right panels, **Figure 4B**). Even more surprising was the much stronger ^13^C2-use after darkening for acetaldehyde formation than compared to incorporation during the light. Moreover, non-stressed plants showed a strong dark burst of acetaldehyde immediately upon light-dark transition, with strong incorporation of ^13^C from ^13^C2-pyruvate. Interestingly, this was not the case in heat stressed plants. Such patterns and strong dark emission of acetaldehyde in heat stressed plants have never been described before.

^13^C-incorporation patterns into mono- and sesquiterpenes were very similar (**Figures 4C, D**). In non-stressed control plants, the C1-atom of pyruvate was not used for biosynthesis of any of these compounds. However, heat stress stimulated use of the C1-atom for terpenoid formation. In contrast to the C1-atom, the C2-atom of pyruvate was used for terpenoid biosynthesis both, in control and heat stressed plants. Notably, heat stressed plants incorporated 3- to 5-times more ^13^C2-pyruvate than plants of the control group (**Figures 4C, D**).

Unfortunately, labeling patterns of methanol were inconclusive, as a reliable peak separation for the mass of the first isotopologue of methanol was not possible. Thus no increase in ^13^C-labeled methanol could be detected, which might be expected as it is a known product from cell wall formation and degradation. However, as data were inconclusive due to these methodological constrains they are not shown.

**Figure 5:**
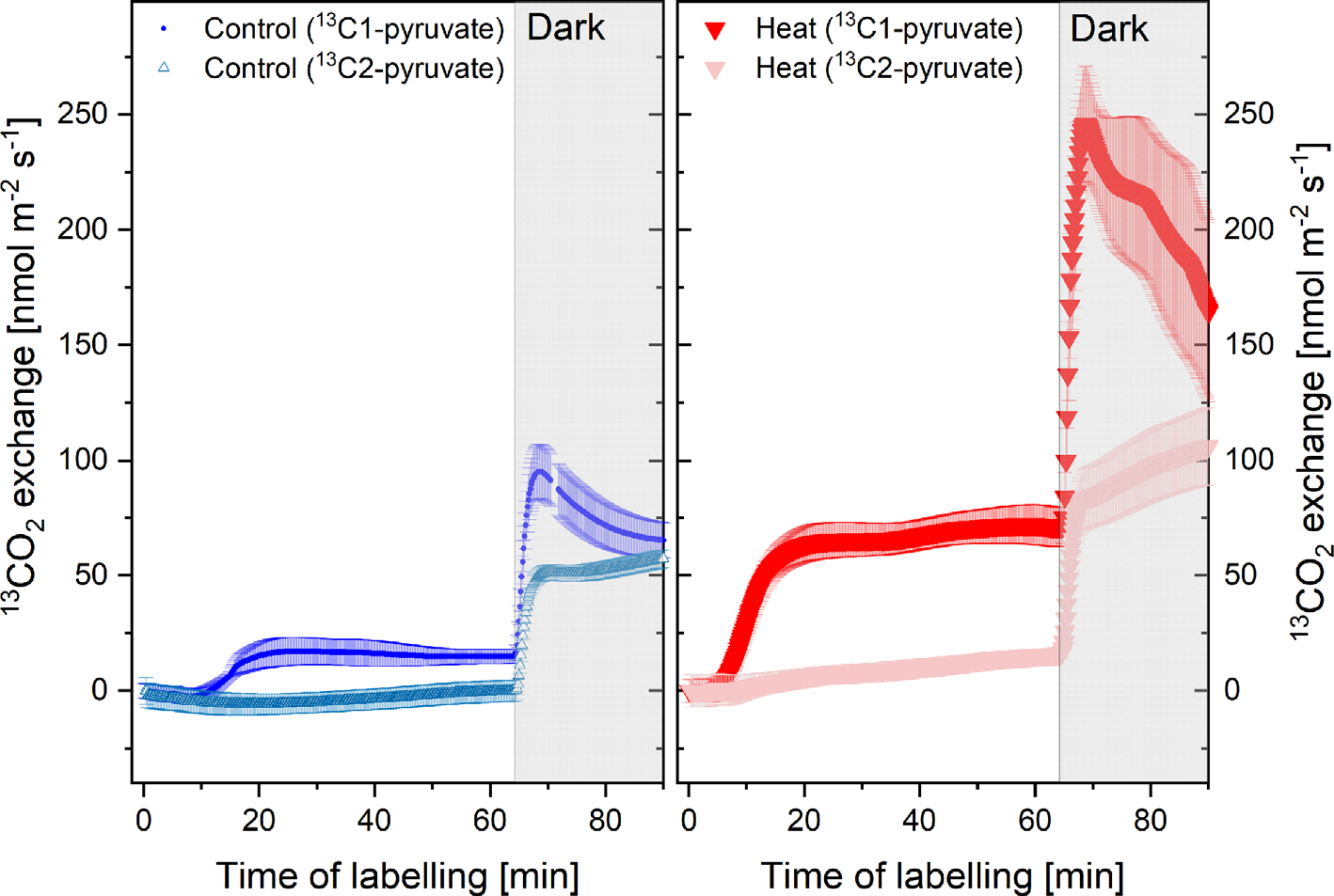
^13^CO_2_ emissions from plants of the control (blue) and heat treatment (red) fed with ^13^C1-pyruvate or ^13^C2-pyruvate for 1 h in the light and 30 min in the dark (shaded area). N= 4–8, standard errors are given. The ^13^CO_2_ flux is given in nmol m^-2^ s^-1^; for calculation see material and methods. Measurements were conducted simultaneously with volatile organic compounds (VOCs) emissions shown in **Figure 4**.

Simultaneous measurements of the isotopic composition of emitted CO_2_ enabled us to follow the release of ^13^CO_2_ from the ^13^C-pyruvate labeled plants. As expected, application of ^13^C1-pyruvate resulted in strong release of ^13^CO_2_ into the atmosphere due to decarboxylation of the C1 position (**Figure 5**). Importantly, heat stress triggered enhanced release of ^13^CO_2_ both under light and dark conditions. In contrast, if ^13^C2-pyruvate was applied, ^13^CO_2_ release from control plants was negligible. Even under heat stress, only small amounts of ^13^CO_2_ were emitted in the light. These results indicate that the pyruvate fed to the plants was not fully decarboxylated in metabolic pathways, i.e., it was not fully respired in the light. Noteworthy, day time CO_2_-emissions from the C1-position of pyruvate more than tripled during heat stress, and so did light-enhanced pulse of dark-respiration during light-dark transitions (**Figure 5**), indicating that these processes were strongly upregulated during heat stress.

## Discussion

Regulation of plant internal carbon allocation is still poorly understood, particularly in response to severe environmental stresses. Here we investigate the impact of a 38°C heat wave on carbon allocation among primary and secondary metabolism in the Mediterranean species *H. halimifolium*. We applied position-specific ^13^C-pyruvate to trace the fate of the ^13^C-atom at the metabolic branching points linking VOC synthesis with respiratory pathways. We could confirm our hypothesis that in spite of a negative carbon balance under severe heat stress (i) enhanced carbon allocation into *de novo* synthesis of particular VOCs occurred even though total terpenoid emissions declined in *H. halimifolium* under prolonged heat stress and (ii) that it was associated with enhanced CO_2_ losses in the light, as discussed below in detail.

Continuous day/night exposure to heat stress over ten days induced highly dynamic responses in *H. halimifolium*: while net photosynthesis significantly declined, respiratory CO_2_ release during both, day and night, markedly increased. Simultaneously, a strong pulse of VOC emissions occurred during the first days (**Figure 3**). Sudden exposure to elevated temperatures exceeded the acclimation potential of *H. halimifolium* and net photosynthesis was further impaired with increasing stress duration, exhibiting a reduced efficiency of photochemical energy conversion of PSII. Elevated respiratory CO_2_ losses affected the net carbon balance of the branches, which turned negative already from day 3 onwards, i.e., leaves suffered from a net carbon loss as nocturnal CO_2_ emissions exceeded diurnal CO_2_ uptake by 78% (**Figure 2**).

Even though plants were well watered, transpiration did not increase to an extend which would help facilitate the dissipation of latent heat (Loreto and Schnitzler, 2010), since stomatal conductance declined during the heat treatment, probably as response to lower relative air humidity in the cuvettes and/or high ci. On the one hand, *H. halimifolium* is a Mediterranean species, well adapted to seasonally hot and dry environments with strong evolutionary pressure to control excessive water loss during these stress periods (Diaz Barradas et al., 1999; Werner et al., 2010; Wegener et al., 2015b). On the other hand, reduced net assimilation and high day respiration and subsequent increased ci values might have counterbalanced the need to open stomata. Even though 38°C can be frequently reached in the Mediterranean summer, continuous exposure to 38°C during day and night might have impaired the photosynthetic machinery as suggested from declined photochemical efficiency.

Day and night-time temperatures were kept constant during the heat wave to allow the assessment of VOC fluxes as well as day and night respiration at the same temperature. However, it must be noted that this implies a larger increase in night-time temperatures during the heat treatment, and thus nocturnal metabolism might have been more strongly affected. This may at least partially explain the significant increase in nocturnal respiration (**Figure 1**). Such an increase in respiration rates under elevated night-time temperatures has been reported in many species, including the Mediterranean shrubs *Heteromeles arbutifolia* and *Lepechinia fragans* (Villar et al., 1995), and many crops, e.g., soybean (Bunce, 2005), rice (Kanno and Makino, 2010; Mohammed et al., 2013) and cotton (Loka and Oosterhuis, 2010). However, even though there is evidence that night-time temperatures may be of particular importance, contrasting effects have been observed (Atkin et al., 2005). For example, night temperatures between 28 and 42.5 °C resulted in substantially increased respiration rates in cotton (Salvucci and Crafts-Brandner, 2004). Therefore, high night temperatures have even been considered one of the main environmental factors contributing to lowered cotton yields (Loka and Oosterhuis, 2010). In contrast, nocturnal temperature increase from 17 to 34°C lead to only minor increases in respiration in rapidly growing soybean, lettuce, and tomato and no effect on cumulative carbon gain was found even after 20 d of treatment (Frantz et al., 2004). These contrasting findings indicate that further research on the impact of nocturnal temperature on the carbon balance of plants is needed. This is particularly relevant in the light of global warming, where night temperatures are predicted to increase faster than day temperatures in several parts of the world (IPCC, 2014). However, while the thermal sensitivity of night respiration can potentially reduce biomass in a warmer climate, acclimation processes have to be taken into account at longer time scales (Atkin and Tjoelker, 2003; Peraudeau et al., 2015).

High temperatures can significantly inhibit photosynthetic efficiency in multiple ways, e.g., through impacts on electron transport activity, or changed ratio of Rubiscos oxygenase/carboxylase activity resulting in higher photorespiration and/or reduced efficiency of Rubisco activase (Havaux, 1993; Law and CraftsBrandner, 1999; Salvucci and Crafts-Brandner, 2004; Carmo-Silva et al., 2015). In the present study, this was reflected in declined maximum quantum yield of photosystem II to 0.570 ± 0.12 after 10 days of heat.

Whereas several studies demonstrated the effects of heat on isoprene emission (Sharkey et al., 2008; Pollastri et al., 2014; Niinemets and Sun, 2015; Bamberger et al., 2017), less information is available on the dynamic response of other VOC fluxes toward prolonged heat stress (Fares et al., 2011; Kleist et al., 2012; Kask et al., 2016). *H. halimifolium* is not a strong isoprene emitter (Yáñez-Serrano et al., 2018), similar to what has been reported for other members of the Cistaceae family (Kesselmeier and Staudt, 1999; Bracho-Nunez et al., 2013). It exhibits a diverse blend of different VOCs, in particular, methyl acetate (Jardine et al., 2014), mono-, sesqui- and diterpenes (Yáñez-Serrano et al., 2018) and a variety of oxygenated and aromatic VOCs (Fasbender et al., 2018). Here we focus on the compounds showing a strong response to heat, namely methyl acetate, acetaldehyde, methanol, as well as mono- and sesquiterpenes and compare these with similar observations from literature.

### Dynamic Response of Oxygenated VOC Emissions to Heat Stress

We observed a marked increase in methyl acetate, methanol and acetaldehyde emissions upon heat stress. However, in contrast to terpenoids, none of these compounds showed an immediate increase upon rising temperatures and strongest emissions occurred between day 2 and 3. Thus a direct physical effect, such as higher volatility due to increased temperature, cannot be the reason for such pattern. Since emission of these compounds increased when the carbon balance turned negative, biosynthesis might be related to a metabolic readjustment of cellular pathways in response to stress. The fast ^13^C-incorporation of ^13^C2-pyruvate into methyl acetate and acetaldehyde, indicates already abundant enzyme activities to ensure fast biosynthesis of these compounds (Fasbender et al., 2018). Enhanced emission of oxygenated VOCs was particularly significant during the morning bursts (e.g., for methanol) when stomata opened upon illumination (**Figure 3**), suggesting that these emissions are under tight stomatal regulation. Such polar compounds accumulate within leaf internal aqueous phases at night (Kreuzwieser, 1999a; Jardine et al., 2014); hence, the extent of morning bursts is directly associated with the compounds’ ability to build up liquid pools during night (Niinemets and Reichstein, 2003), which are then released when stomata are opening in the morning. In contradiction to these observations, in the present work, heat stressed plants showed high emissions at night. Considering the slightly increased nocturnal transpiration, this might indicate that heat stress either prevented full nocturnal stomatal closure or it induced enhanced biosynthesis of these oxygenated compounds, raising leaf internal VOC partial pressures and inducing diffusion even through mostly closed stomata (Kreuzwieser et al., 1999b).

**Figure 6:**
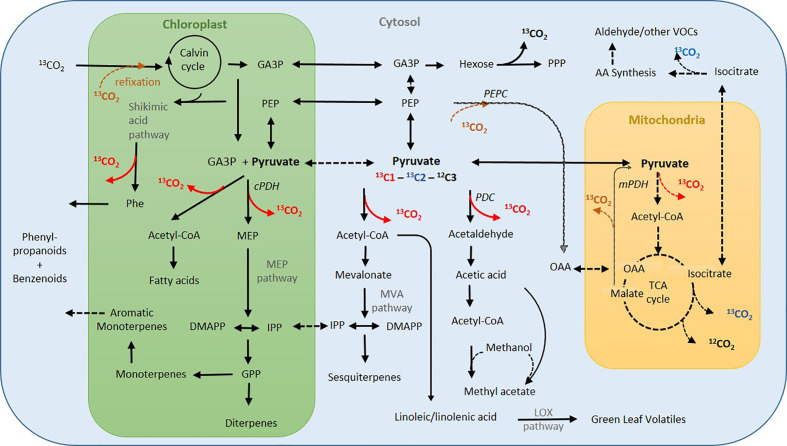
Simplified metabolic network indicating major pathways of volatile organic compound (VOC) synthesis and associated CO_2_ decarboxylation processes in the light. Dark red arrows indicate potential sources of upregulated day CO_2_ flux during heat stress (from C1-pyruvate), blue denotes CO_2_ decarboxylation from C2-pyruvate. Dashed lines: low flux in the heat and under light. Pathway names in grey, enzymes in italic. Acetyl-CoA, acetyl coenzyme-A; AA, amino acids; DMAPP, dimethylallyl diphosphate; GA3P, glyceraldehyde-3-phosphate; GPP, geranyl diphosphate; GPP, geranyl diphosphate; IPP, isopentenyl pyrophosphate; LOX, lipoxygenase; MEP, MEP,2-C-methyl-D-erythritol 4-phosphate; MVA, mevalonate; OAA, oxaloacetate; PDH, pyruvate dehydrogenase; mPDH, mitochondrial PDH; cPDH, chloroplastic PDH; PDC, pyruvate decarboxylase; PEP, phosphoenolpyruvate; PEPC, PEP carboxylase; Phe, phenylalanine; PPP, pentose phosphate pathway; TCA, tricarboxylic acid cycle. Information partially derived from (Dudareva et al., 2006; Dudareva et al., 2013; Tcherkez et al., 2017; Fasbender et al., 2018).

*H. halimifolium* is a strong methyl acetate emitter (Jardine et al., 2014) with high intra-specific variability (Fasbender et al., 2018, **Figure 3**). So far, not many species are characterised as strong methyl acetate emitters (Davison et al., 2007; Jardine et al., 2010), though *H. halimifolium* emits methyl acetate at ca. 10-fold higher rates than monoterpenes. Here, we demonstrate for the first time that methyl acetate emissions markedly increased with heat stress (**Figure 3**). It is assumed that methyl acetate biosynthesis starts from pyruvate, which is decarboxylated in a first reaction to produce acetaldehyde that is further oxidized to acetate (**Figure 6**). Either acetate or its activated form acetyl-CoA reacts with methanol to eventually form methyl acetate. Because of this assumed pathway, we expected similar emission patterns for methyl acetate and acetaldehyde, which were partially observed. Both compounds showed enhanced emission in heat stressed plants, particularly during night-time. To our knowledge, this is the first report showing a heat dependent, rise in nocturnal acetaldehyde emission which exceeded daytime fluxes by far. Though we did not label the plants during the night, darkening the leaves for 30 min revealed a substantial increase in *de novo* synthesis by ^13^C-incorporation of ^13^C2-pyruvate (**Figure 4**). This suggest that night-time emissions were also driven by *de novo* synthesis. This may have fostered the morning burst of acetaldehyde which were more than one order of magnitude higher in heat stressed plants than in the control group. Further research is needed to investigate the role of nocturnal acetaldehyde synthesis during prolonged heat stress in different species. Similar to our results, Behnke et al. (2013) observed heat-induced acetaldehyde production (during 4 h 40°C) in excised poplar leaves. However, they suggested that acetaldehyde might originate from fatty acid peroxidation reactions after accumulation of ROS (Jardine et al., 2009) during membrane breakage in damaged tissue (Fall et al., 1999; Loreto et al., 2006; Brilli et al., 2011). Indeed, heat-induced membrane dysfunction can result in the release of various compounds such as aldehydes or ethane (Nanaiah and Anderson, 1992). However, we observed high emission in intact branches of well-watered plants exposed to heat, and, thus, desiccation effects can be excluded. Though we cannot exclude direct effects of heat or ROS inducing membrane dysfunction, strong ^13^C-incorporation from ^13^C2-labeled pyruvate provides clear evidence that acetaldehyde bursts also derive from substantial *de novo* synthesis. Although the source of acetaldehyde emission is still a matter of debate (Graus et al., 2004), it seems to be induced by stress (Loreto and Schnitzler, 2010), e.g., during wounding or desiccation under high temperature (Holzinger et al., 2000; Loreto et al., 2006; Brilli et al., 2011) and after root flooding (Kreuzwieser, 1999a; Kreuzwieser et al., 2001). Morning bursts could be indicative of root produced acetaldehyde transported to the leaves when transpiration increases after sunrise (Kreuzwieser et al., 1999b).

Upon darkening, we found another burst of acetaldehyde during light‐to‐dark transition under control-conditions (**Figure 4**, blue lines), which has often been described (Holzinger et al., 2000; Karl et al., 2002; Brilli et al., 2011; Ghirardo et al., 2011; Jardine et al., 2012; Jud et al., 2016). Pyruvate labeling indicated that the dark-burst of acetaldehyde originated from *de novo* synthesis from the acetyl-CoA moiety of pyruvate (**Figure 6**). In agreement with Jud et al. (2016) and Ghirardo et al. (2011), dark burst of acetaldehyde was reduced or even absent in stress‐affected plants (see **Figure 4B**, red lines).

As mentioned above, biosynthesis of methyl acetate may also depend on the availability of methanol as a precursor. Heat strongly enhanced methanol emission when temperatures were stepwise increased, though with a time-lag of a few hours (**Figure 3**), and highest emissions occurred during the morning bursts on days 2 and 3 of the heat wave. Methanol is known to be predominantly emitted from degradation (and formation) of cell wall pectins, e.g., during senescence and leaf abscission (Harriman et al., 1991; Loreto and Schnitzler, 2010; Niederbacher et al., 2015). To a minor extent, methanol emissions might also originate from protein methyltransferase and protein repair reactions (Kreuzwieser et al., 1999b), which could be enhanced in response to stress. Methanol emissions are also found in response to mechanical wounding or other stresses due to damage of the cell wall (Dorokhov et al., 2018), or with increasing temperatures (Schade et al., 2011). Interestingly, emissions from wounded plants can induce defence reactions in intact leaves of the same and neighbouring plants, by activating methanol-inducible genes that regulate plant resistance to biotic and abiotic factors (Dorokhov et al., 2018). Unfortunately, we did not find a clear labeling signal for methanol due to difficulties in peak separation for the ^13^C-isotopologue of methanol. Ghirardo et al. (2011) reported significant ^13^C-labeling of methanol emitted from mature leaves and apices of shoots if plants were fed with ^13^C-Glc, but not with ^13^CO_2_. However, in absence of further evidence, we suggest that cell wall degradation might be the primary source of large methanol emission, particularly in highly impacted plants during heat stress.

### Dynamic Response of Terpenoid Emissions to Heat Stress

In contrast to oxygenated VOCs, both mono- and sesquiterpene emissions increased instantaneously with higher temperatures probably due to increased volatility of these terpenoids. They exhibited highest emissions during the first 48 h of heat exposure and thereafter declined. Isoprene and monoterpene emissions are expected to stabilize thylakoid membranes and proteins, thereby enhancing the plant’s thermotolerance (Singsaas et al., 1997; Singsaas, 2000; Sharkey, 2005). Biophysical evidence indicates that isoprene improves the integrity and functionality of the thylakoid membranes at high temperature, i.e., though stabilisation of the ordered arrays of light harvesting complex and photosystem II in the thylakoid grana (Velikova et al., 2012). Further mechanisms are protection of the thylakoid membrane against leakiness to maintain the proton-motive force and enhance the efficient primary photochemistry of PSII (Velikova et al., 2011). Recent studies have shown that this is most likely mediated by isoprene binding to proteins, and by modulating the dynamics of thylakoid-embedded proteins that affect membrane stabilization (Harvey et al., 2015).

However, here it is evident that the sudden increase by +13°C obviously exceeded the capacity of heat acclimation in *H. halimifolium*, even though typical stress markers like green leaf volatiles were not emitted in large amounts (data not shown). Interestingly, even though the heat wave might have depleted the terpene pools within the first 48 h, ^13^C2-pyruvate incorporation into both, mono- and sesquiterpenes (**Figures 4C, D**) clearly indicated *de novo* synthesis. This finding corroborates that terpene emissions in *H. halimifolium* are driven by both, temperature dependent release of stored compounds (i.e., storage pools for β-caryophyllene and farnesene, see Yáñez-Serrano et al., 2018) and *de novo* biosynthesis (Fasbender et al., 2018). Remarkably, even though the absolute emission rates of monoterpenes and particularly sesquiterpenes declined after 10-days of heat (**Figure 3**), the proportion of *de novo* synthesis of mono- and sesquiterpenes was strongly enhanced during heat (**Figure 4**) despite limited carbon availability. This might be underlining the importance of carbon investment into these compounds in order to enhance stress tolerance in plants.

Pyruvate is a precursor for both, monoterpenes and sesquiterpenes. However, whereas monoterpenes are generally synthesised *via* the chloroplastic methylerythrol-phosphate (MEP) pathway, sesquiterpenes are produced *via* the cytosolic mevalonate (MVA) pathway (Dudareva et al., 2006). Therefore, for the use of the applied labeled ^13^C-pyruvate in the MEP pathway it needs to be transported into this organelle. It has been reported that pyruvate can be shuttled into the chloroplast either directly *via* a pyruvate transporter, after conversion to PEP *via* a PEP transporter , *via* GA3P, or *via* a malate shuttle (Dudareva et al., 2006; Linka and Weber, 2010; Dudareva et al., 2013; Souza et al., 2018). In both pathways, the C1 of pyruvate is then decarboxylated and released as CO_2_ and only the C2-C3 moiety is further used for biosynthesis (**Figure 6**). It was, therefore, unexpected to find incorporation of the ^13^C1 of pyruvate in both terpene classes in heat stressed plants. We could assume that photosynthetic re-fixation of day respired ^13^CO_2_ occurred; in consistence with this view, incorporation of the ^13^C1 of pyruvate was more pronounced in mono- than in sesquiterpenes, since monoterpene biosynthesis is directly fuelled from fresh photosynthates formed in the chloroplasts. The ^13^C1-incorporation into sesquiterpenes, however, requires export of ^13^C-labeled metabolites from the chloroplast. This can be either an export of labeled triose phosphates or it points toward substantial crosstalk between MEP- and MVA-pathway (Souza et al., 2018). The fact that none of the cytosolic oxygenated VOCs did show significant incorporation of ^13^C1-label could be interpreted as an export of IPP *via* an postulated IPP transporter (Arigoni et al., 1997; Hemmerlin et al., 2003). However, recent work has shown little evidence of a large exchange of IPP (Rasulov et al., 2018; Souza et al., 2018).

Moreover, it must be denoted that a strong imprint of ^13^CO_2_-refixation would result in an equal distribution of ^13^C-label at all carbon positions. As triose phosphates are exported from the Calvin cycle and fuel cytosolic processes, a similar labeling pattern in all *de novo* synthesised molecules would be expected, if re-fixation would play a dominant role. Furthermore, ^13^CO_2_ decarboxylation was significantly higher from ^13^C1-pyruvate than from ^13^C2-pyruvate (**Figure 5**), and thus cells were exposed to higher ^13^CO_2_ concentrations from ^13^C1-pyruvate decarboxylation. Therefore, the impact of refixation would be expected to be higher from ^13^C1-pyruvate feeding, which was clearly not the case (Yáñez-Serrano et al., 2019) (**Figure 4**). It has recently been shown that there is substantial elasticity in the MEP pathway, strongly related to the availability of reducing power and ATP from photochemical reactions (Rasulov et al., 2015; Rasulov et al., 2018). Souza et al. (2018) proposed that depending on the availability photochemical energy supply and the limitations imposed by either Rubisco activity or RuBP regeneration (under high and low CO_2_ and temperature) multiple alternative carbon sources can fuel the MEP pathway. They conclude that the use of alternative carbon sources is greater under photosynthesis-limiting conditions, which is in line with our results, showing that the use of cytosolic pyruvate for *de novo* synthesis is strongly enhanced under heat stress and limited photosynthetic carbon fixation (Yáñez-Serrano et al., 2019) (**Figure 4**). However, there could also be alternative pathways which are currently not understood.

### Day and Dark Respiration

The strong increase of dark respiration in response to heat was mirrored by enhanced decarboxylation during day (**Figure 5**). Interestingly, this enhanced metabolic day respiration was not evenly fuelled from all C-positions of the pyruvate molecule: in the light, CO_2_ was mostly emitted from the C1-position of pyruvate and only upon darkening, substantial decarboxylation of the C2 position, most probably from up-regulation of the TCA-cycle in the mitochondria, occurred. The low decarboxylation of the C2 position of pyruvate in the light indicates down-regulation of the TCA-cycle activity (Tcherkez et al., 2005; Werner et al., 2009). It is assumed that the TCA-cycle undergoes a major re-organization during illumination (Sweetlove et al., 2010; Sweetlove et al., 2013), in order to supply carbon skeletons, e.g., for amino acid synthesis (Tcherkez et al., 2017). Thus, high decarboxylation from the C1-position of pyruvate occurred when carbon skeletons are used for processes such as synthesis of many secondary compounds including fatty acids and VOCs. Remarkably, during heat stress we observed that such uneven decarboxylation of the C1 and C2 of pyruvate was even enhanced as heat caused a three-fold increase in decarboxylation of C1: this unexpected finding of low C2-pyruvate decarboxylation led us hypothesize that the CO_2_ released from heat stressed plants in the light is mainly derived from cytosolic and chloroplastic reactions and not from the TCA-cycle (**Figure 6**). In accordance with studies suggesting that pyruvate uptake into mitochondria, as well as the activities of mitochondrial pyruvate dehydrogenase, malic enzyme, isocitrate dehydrogenase and 2-ketoglutarate dehydrogenase of the TCA cycle are light inhibited (Tovar-Mendez et al., 2003; Tcherkez et al., 2005; Sweetlove et al., 2010; Tcherkez et al., 2017). The fact that very low decarboxylation of C2-pyruvate in the light was observed might be an indication, that under extreme heat stress and limiting carbon availability, also the non-cyclic pathways of the TCA-cycle, which supplement the organic carbon pool (Tcherkez et al., 2017), might have been down-regulated. However, further work is needed to substantiate functioning of the TCA cycle under prolonged heat.

It has been hypothesized that high day respiration may compete with isoprene biosynthesis for pyruvate or PEP, because decreased isoprene emissions were found in heat-acclimated plants or in plants grown under elevated CO_2_ (Loreto et al., 2007). Other work suggests that the partitioning of PEP and pyruvate between mitochondrial respiration and chloroplastic VOC synthesis is controlled in a way that retains the balance in substrate demand (Loreto et al., 2007). However, our position-specific labeling clearly demonstrates that VOC synthesis contributes, rather than competes, to enhanced daytime CO_2_ emissions due to metabolic partitioning of the pyruvate precursor (CO_2_-releasing pathways during VOC-synthesis under heat stress are indicated in **Figure 6**): the synthesis of the universal precursors for terpenoids, isopentenyl pyrophosphate (IPP) and dimethylallyl diphosphate (DMAPP), requires three acetyl-CoA moieties in the MVA pathway. These are provided by decarboxylation of three pyruvate molecules and, thus, results in the release of 3 CO_2_ molecules. Thus, synthesis of one mole sesquiterpenes (i.e., 15 carbon skeletons) *via* the MVA pathway is associated with release of 9 mol CO_2_. In contrast, in the MEP pathway only one mole CO_2_ is released per mole IPP as after decarboxylation pyruvate reacts with the C1-aldehyde group of GA3P derived from the Calvin cycle (Rohmer et al., 1996; Arigoni et al., 1997). Hence, monoterpene synthesis in the MEP pathway should provide a lower ratio of CO_2_ decarboxylation and, therefore a lower incorporation of ^13^C2-label compared to precursors synthesised *via* the MVA pathway. However, as pointed out above, we found similar labeling patterns of monoterpenes and sesquiterpenes (**Figure 4**), which might be another strong indication for substantial crosstalk between MVA and MEP pathway as discussed above.

## Conclusions

In summary, these results show that the sudden exposure to a heat wave can exceed the acclimation potential even of a Mediterranean species, resulting in a marked depression of net photosynthesis, and enhanced respiratory losses inducing a negative carbon balance in plants. Nevertheless, marked investment into *de novo* synthesis of several VOC was found, even at the expense of further respiratory losses in the light. Given the fact that plants invest carbon in a plethora of VOCs even under stress conditions is a clear indication for their importance for plant protection and survival, though for many poorly studied compounds their physiological role in stress protection is not yet fully resolved. Moreover, the fact that extreme heat waves may shift plants into a negative carbon balance and that assimilation at temperatures exceeding the optimal condition is more inhibited than respiration, pose important interrogatives on the role of plant carbon balance in future scenarios of increasing temperatures and extreme events (Loreto et al., 2007).

## Data Availability Statement

The datasets generated for this study are available on request to the corresponding author.

## Author Contributions

CW design the research. LF and KR planned and conducted the experiments. AY-S and JK performed the data analysis. CW and JK interpreted data. CW wrote the manuscript with substantial input of JK, and revision by all authors. All authors contributed to the article and approved the submitted version.

## Funding

This work was financed by the ERC consolidator grant of CW (VOCO_2_; 647008).

## Conflict of Interest

The authors declare that the research was conducted in the absence of any commercial or financial relationships that could be construed as a potential conflict of interest.
